# Safety, structure and function five years after hESC-RPE patch transplantation in acute neovascular AMD with submacular haemorrhage

**DOI:** 10.1007/s00417-024-06463-4

**Published:** 2024-04-05

**Authors:** Taha Soomro, Odysseus Georgiadis, Peter J. Coffey, Lyndon da Cruz

**Affiliations:** 1 The London Project to Cure Blindness, ORBIT, Institute of Ophthalmology, University College London (UCL), London, UK; 2 NIHR Biomedical Research Centre at Moorfields Eye Hospital NHS Foundation Trust, UCL Institute of Ophthalmology, London, UK; 3 Moorfields Eye Hospital NHS Foundation Trust, London, UK; 4 Center for Stem Cell Biology and Engineering, NRI, UC, Santa Barbara, CA USA

**Keywords:** Retinal pigment epithelium, Neovascular age-related macular degeneration, Sub-macular haemorrhage, Stem cell therapy, Human embryonic stem cells

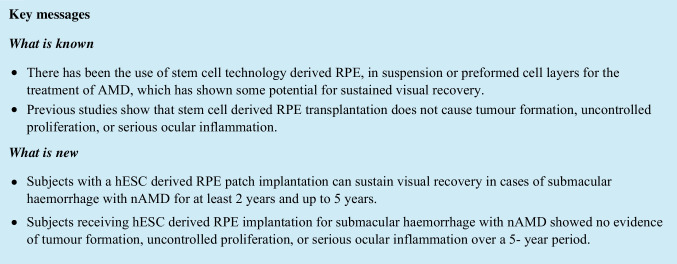



## Introduction

Recent trials in stem-cell derived cell transplantation, for retinal diseases, have suggested the potential for visual recovery [1, 2]. Despite this, concerns about their potential risks remain [3, 4]. Currently, data from these studies in humans has been published to 1 years using human Embryonic Stem Cell derived (hESC) retinal pigment epithelium (RPE) monolayer [1, 2] and 4 years using induced Pluripotent Stem Cells (iPSC) monolayer transplantation [2]. Importantly, none of these have reported evidence of uncontrolled RPE proliferation, tumour formation or major ocular immune reactions [1, 2]. In this brief report, we describe safety, structural and functional outcomes to 5-years for 2 patients following hESC-RPE monolayer transplantation.

## Methods

The study, including patient selection, surgical procedures, derivation of hESC-RPE, production of the patch, clinical investigations and detailed results to 12 months have been previously described [1]. Functional assessment of refracted best corrected visual acuity (ETDRS) was carried out by independent optometrists.

## Results

### Safety

There were 4 serious adverse events (SAEs) during the study. Subject 1 developed a scleral suture related conjunctival erosion which was repaired surgically. Subject 2 developed a peripheral retinal detachment, 2 months after the initial surgery, due to proliferative vitreoretinopathy (PVR). It was repaired with a single operation with retention of silicone for an additional 2 months before removal. The retina remained fully attached to 5-years. The other two SAEs were related to Subject 2 comorbidities with elevated blood sugars related to peri-operative oral steroids, managed medically, and a transient ischaemic attack (TIA) in year 2.

There were no signs of uncontrolled RPE proliferation, ectopic differentiation or tumorigenicity on assessment during the five-year period.

### Function

**Fig. 1:**
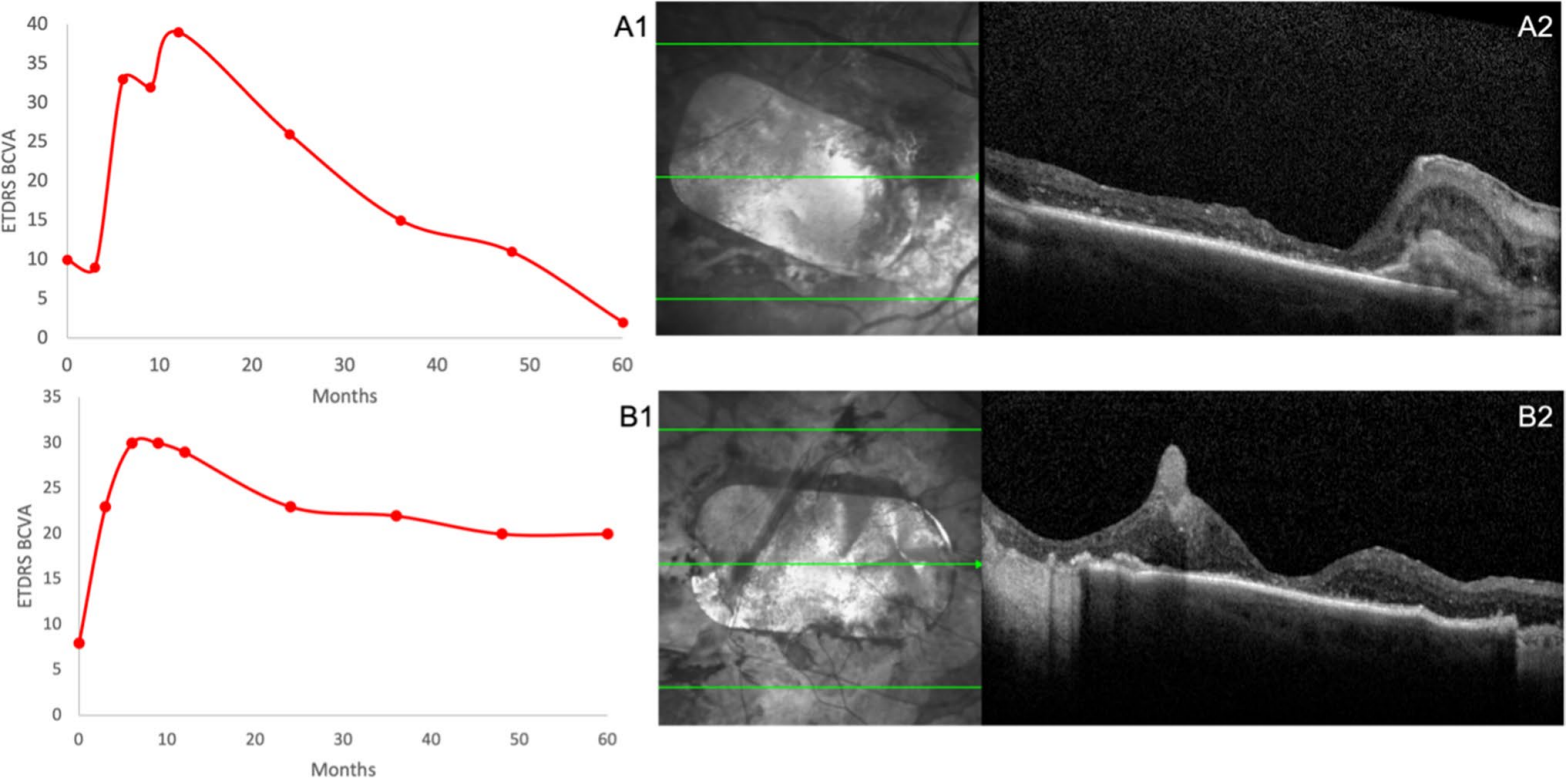
Visual and structural function during the 5-year follow up of both subjects 1(A) and 2(B). (**A1**, **B1**) change in BCVA (ETDRS letters), (**B1**, **B2**) OCT slice through fovea with subretinal hESC patch in situ at 5 years

Subjects 1 and 2 had an improved best corrected visual acuity (BCVA) at 2 years of 16 and 15 letters respectively. For Subject 1 there was a linear reduction from 2 years; reaching baseline at 4 years (11 letters) and below baseline at 5 years (2 letters) (Fig. 1. A1). For Subject 2 the improved BCVA reduced but remained above baseline by 9 letters at 5 years (Fig. 1. A2).

### Structure

**Fig. 2:**
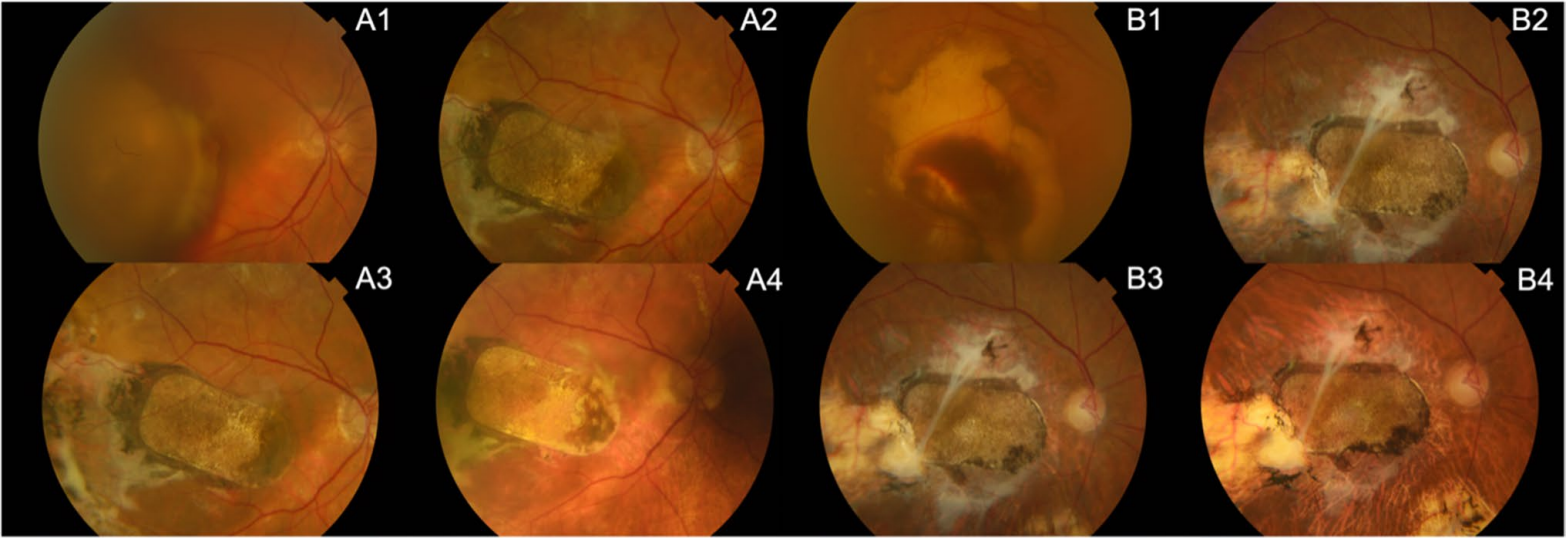
Patch position and pigmentation during the 5-year follow up of both subjects 1(A) and 2(B). (**A1**, **B1**) baseline, pre-operatively, (**A2, B2**) at 12 months post-operatively, (**A3, B3**) at 24 months, (**A4, B4**) at 5 years

SD-OCT showed areas of continuous, double, hyper-reflective lines consistent in thickness and position with the hESC-RPE, and matching areas of pigmentation for both patients at 5 years (Fig. 1. A2 and B2, Fig. 2. A4 and B4). For subject 1 the neurosensory retina was thinned over the patch with no regular architecture at 5 years (Fig. 1. A2). Subject 2 had preservation of some retinal architecture with thinned fovea at year 5. (Figure 1. B2).

## Discussion

We observed patch pigmentation corresponding to OCT signals suggesting persistence of transplanted RPE in both patients at 5 years (Fig. 1. A2 and B2, Fig. 2. A4 and B4). We cannot be certain whether this was because of the surgery, the health of the neuroretina, the function of the choriocapillaris or a low-grade, chronic rejection-related inflammation, as no histology is available.

Sustained BCVA improvement to 2 years for subject 1, and, 5 years for subject 2 was recorded, though it is not possible to attribute the improvement to the RPE transplant alone in the absence of a controls. Improvements in vision have been seen with removal of blood alone [5], but these results suggests that the transplanted RPE may be capable of sustaining retinal function long-term.

In terms of safety, there was no uncontrolled proliferation or tumorigenicity and no clinically evident intraocular inflammation over the five-year period. Both subjects received oral and topical steroids as immunosuppression as described previously [1]. In both subjects epiretinal and sub-retinal fibrosis was seen and in subject 2 a PVR retinal detachment occurred, which did not occur or progress after the 1^st^ 12 months. It is possible that the fibrosis and retinal detachments were related to the transplanted RPE or the underlying neovascular AMD combined with retinal surgery.

## Conclusion

We report findings at 5 years for 2 patients with hESC-RPE sheet transplantation to provide early information, that will assist the preparation of further and more definitive studies in this field, that will begin to answer the many ongoing questions in the field.
